# Developmental co-calibration and therapeutic recalibration: a neurobiopsychosocial model of regulatory path dependence in psychosomatic care

**DOI:** 10.3389/fpsyt.2026.1867979

**Published:** 2026-08-13

**Authors:** Eik Niederlohmann

**Affiliations:** Department of Psychosomatic Medicine and Psychotherapy, Kliniken Erlabrunn, Breitenbrunn, Germany

**Keywords:** developmental co-calibration, regulatory path dependence, regulatory flexibility, interoception, person–environment fit, psychosomatic medicine, psychotherapy process, environmental accommodation

## Abstract

Psychotherapy and psychosomatic medicine require accounts that connect developmental context, embodied regulation, bodily symptoms, and treatment processes without collapsing them into a single causal story. This Hypothesis and Theory article presents developmental co-calibration as an integrative, testable specification rather than a set of novel component mechanisms. It proposes a staged chain in which biological dispositions and current affordances shape regulatory states; states and candidate interoceptive inference processes influence strategy selection; immediate consequences and environmental responses feed back into later states and contexts; and repeated episodes may alter transition probabilities over development. Its model-specific claim is regulatory path dependence: a prespecified distal regulatory history should improve prediction of current state or strategy transitions after accounting for current context, state, stable person characteristics, measurement opportunity, and immediate inertia. This claim differs from current regulatory inflexibility, variability, avoidance, and habit. The cited literatures inform individual links involving developmental plasticity, adversity, allostasis, interoception, regulatory flexibility, camouflaging, and psychotherapy processes; none tests the complete sequence. The framework therefore remains a staged, falsifiable research program. Health implications are separated into disease onset, exacerbation or course, persistent physical symptoms, functional somatic disorders, and multimorbidity; psychogenic causation is not inferred. Therapeutic recalibration is defined primarily as an intervention- or accommodation-associated change in the prespecified distal-history effect when baseline history predicted poorer fit, functional cost, or delayed recovery. Context–strategy–outcome fit and recovery are separate secondary process outcomes; symptom improvement alone does not qualify. State-matched psychotherapy and environmental accommodation are complementary candidate routes. The article specifies competing frameworks, measurable signatures, falsification criteria, and safeguards of medical humility and epistemic dignity.

## 1 Introduction: the unresolved value of regulatory history

Psychotherapy and psychosomatic medicine encounter presentations that cross boundaries maintained by diagnostic systems and institutions. Breathlessness, pain, exhaustion, panic-like arousal, shutdown, social withdrawal, attentional dysregulation, or persistent bodily distress may be described as somatic, affective, behavioral, relational, or neurodevelopmental depending on the observer and setting. These perspectives can each be valid, yet fragmentation changes what is measured, which explanations are considered legitimate, and which interventions become available.

The biopsychosocial model established that biological, psychological, and social dimensions cannot be separated in clinical understanding (1, 2). Developmental systems and cascade approaches already describe reciprocal, probabilistic transactions across levels and time (3, 4). Allostasis and biological embedding describe cumulative physiological costs of adaptation (5–8), while the Adaptive Calibration Model (ACM) proposes developmentally calibrated patterns of stress responsivity (9). Regulatory-flexibility and person–environment-fit frameworks further show that no strategy is adaptive independently of context, repertoire, feedback, and current ecology (10, 11). These are inherited premises, not discoveries of the present model.

The unresolved question is narrower: Does prior regulatory history explain current regulatory transitions after present conditions have been measured? Two people may appear equally inflexible now, yet only one may show an additional, replicable effect of earlier strategy–context–feedback sequences on what happens next. Developmental co-calibration is proposed to organize this question across within-episode and developmental timescales. The model does not propose novel component mechanisms. Its incremental contribution is a developmental and history-dependent specification of when contextually useful regulatory strategies become less context-sensitive, harder to revise, and more costly—and how state-matched psychotherapy and environmental accommodation could testably modify a prespecified history effect associated with poor fit or delayed recovery.

An integrative theory must specify units, temporal order, alternative explanations, and disconfirming observations. Here, the units are regulatory states, regulatory strategies, transitions, and context-coded feedback; the test is incremental transition prediction. Clinical intelligibility is not empirical confirmation.

This article has three aims. First, it defines regulatory state, strategy, flexibility, path dependence, and therapeutic recalibration in discriminable and measurable terms. Second, it presents a staged hypothesis chain, makes overlap with the closest frameworks explicit, and derives predictions that could fail. Third, it translates the model into general psychotherapy-process requirements while retaining medical humility, outcome specificity, and neurodiversity-affirmative boundary conditions. The theory earns incremental value only if history-sensitive predictions outperform models based on current state, context, flexibility, diagnosis, and symptom severity.

## 2 From explanatory levels to recursive regulatory loops

Neurobiopsychosocial here denotes embodied and socially situated regulation without privileging neural language. Candidate processes include autonomic regulation, interoception, affective learning, action selection, immune signaling, and interpersonal or institutional feedback. The model-specific addition is a defined history term, not recursive coupling itself.

A regulatory state is a time-varying configuration of arousal, affect, attention, action readiness, and clinically relevant bodily estimates. A regulatory strategy is an observable or reportable action or process used to influence internal state, behavior, interpersonal conditions, or environmental demands. State and strategy must therefore be measured separately: anxiety is not a strategy, withdrawal is not necessarily a state, and symptom improvement is neither. Context-conditioned preference is used only as a descriptive bridge for an increased probability of selecting or maintaining a strategy within a prespecified context class.

Regulatory flexibility is imported from existing literature and denotes the coordinated capacity to detect contextual demands, access a usable repertoire, and maintain or switch strategies in response to feedback (10). Greater variability does not necessarily indicate greater flexibility; daily-life research can separate how often strategies vary from whether their use is context-sensitive or beneficial (12, 13). Psychological or emotion regulation inflexibility describes a current pattern. Regulatory path dependence makes an additional historical claim and is defined in Section 4. Habit or automaticity may contribute to that history effect but is not synonymous with it (14).

Interoceptive predictive processing offers a candidate account of part of the within-episode loop. A current regulatory state may reflect priors about bodily conditions, precision-weighted afferent signals, and action-dependent sampling that changes which signals are encountered. Repeated mismatch or strategy use could, in principle, alter expectations, precision allocation, or sampling. However, adult interoceptive models do not establish this developmental pathway (15, 16). Interoception is multidimensional, develops across childhood and adolescence, and is measured heterogeneously in younger populations (17, 18). Precision weighting, altered priors, and afferent coding are therefore mechanistic candidates for specified links, not observed mediators of the full co-calibration chain.

The model uses three model-specific terms: developmental co-calibration, regulatory path dependence, and therapeutic recalibration. Regulatory state, strategy, flexibility, habit, allostasis, person–environment fit, and active interoceptive inference are imported. Context-conditioned preference and costly adaptation are descriptive bridges. Epistemic dignity, medical humility, differential formulation, and therapy-school neutrality are ethical-methodological safeguards rather than mechanisms. **Table 1** fixes these distinctions and the corresponding measurable signatures.

**Table 1:** Construct-to-estimand matrix.

Construct	Status	Working definition	Unit/timescale	Measurable signature/estimand	Discriminant comparator	What would not count
Regulatory strategy	Literature	Observable or reportable action/process used to modulate internal state, behavior, or environmental demands.	Episode; seconds–days	Strategy category, intensity, or duration after a defined state/context.	State; outcome	Feeling anxious; symptom improvement alone.
Regulatory state	Literature	Time-varying configuration of arousal, affect, attention, action readiness, and relevant interoceptive estimates.	Moment/episode	Multimodal state; transition probability; dwell time.	Strategy; stable trait	Diagnosis or trait scale used as the state.
Context-conditioned preference	Descriptive bridge	Higher probability of selecting or maintaining a strategy in a prespecified context class.	Repeated episodes	P(strategy | context,state); context-specific selection slope.	Habit; flexibility	Frequent strategy use without a context definition.
Regulatory flexibility	Literature	Context sensitivity, usable repertoire, and feedback-responsive maintenance or switching.	Episodes–weeks	Context–strategy fit; appropriate switching/maintenance; outcome-contingent updating.	Variability; path dependence	More switches or strategies regardless of fit.
Regulatory path dependence	Model-specific	Distal-history predictive contribution to current state/strategy transitions after current context/state, stable characteristics, and immediate lag.	Weeks–years, measured intensively	β_history; incremental/out-of-sample gain from M2 to M3; optional history × mismatch.	Current rigidity; inertia; habit	High autocorrelation, adversity, or inflexibility alone.
Therapeutic recalibration	Model-specific	Intervention-/accommodation-associated change in prespecified β_history under the baseline risk condition.	Treatment phase and follow-up	Primary: Δβ_history; secondary: context fit and recovery; clinical outcomes separate.	Response/remission	Symptom decrease without Δβ_history; unconditioned reduction of any history effect.

Constructs are separated even when measured within the same intensive longitudinal design. No single questionnaire, task, or physiological signal directly measures regulatory path dependence or therapeutic recalibration.

Measurement opportunity is part of the definition. A strategy cannot be judged unavailable if the relevant context never occurred, and a transition cannot be estimated if assessments are too sparse for its timescale. Studies should therefore record exposure opportunities, sampling intervals, missingness, and whether reported nonuse reflects choice, constraint, safety, or lack of access. Multi-informant and multimodal data may improve triangulation, but no modality automatically outranks the person’s report or converts a descriptive association into a mechanism.

## 3 Relation to existing frameworks and incremental contribution

Developmental co-calibration does not replace adjacent theories. Developmental systems and cascades already model reciprocal development; the Adaptive Calibration Model addresses calibrated stress responsivity; regulatory flexibility addresses context-sensitive switching or maintenance; person–environment fit addresses current configuration; habit addresses cue-dependent automaticity; active-inference accounts address state estimation and action; and process-based therapy targets modifiable processes (3, 4, 9–11, 14, 15, 19). **Table 2** identifies what is imported and what remains testable.

**Table 2:** Comparison with adjacent frameworks.

Framework	Established contribution imported	Remaining question	Incremental specification	Discriminating prediction
Biopsychosocial models (1, 2)	Multilevel determinants and clinical integration.	Often broad regarding temporal dynamics and estimands.	A staged history parameter within reciprocal loops.	History improves transitions beyond current multilevel variables.
Probabilistic epigenesis/developmental systems (3)	Reciprocal probabilistic coaction across levels.	Does not specify the present regulatory estimand or therapy test.	Prespecified strategy/state transitions plus intervention-associated parameter change.	β_history and Δβ_history replicate in the predicted direction under defined controls.
Developmental cascades (4)	Cumulative, bidirectional cross-domain effects.	May remain domain-general or outcome-level.	Separates within-episode feedback from distal history effects.	Sequence predicts transitions after lag and traits.
Adaptive Calibration Model (9)	Developmental calibration of stress responsivity.	Focuses on responsivity rather than joint strategy–feedback history and clinical parameter change.	Added value of strategy/environmental-feedback history under mismatch.	History adds beyond responsivity plus current ecology/fit.
Regulatory flexibility (10)	Context sensitivity, repertoire, and feedback-responsive switching/maintenance.	Need not posit a distal developmental-history parameter.	Separates current flexibility from binding to distal history.	History adds beyond measured flexibility.
Person–environment fit (11)	Outcomes depend on current person–environment configuration.	Current fit need not explain effects of earlier sequences.	History is incremental only beyond current fit; accommodation can change fit.	M3/M4 outperform a current-fit model.
Active/interoceptive inference (15, 16)	State estimation, prediction error, precision weighting, and action.	Can explain momentary regulation without the developmental history estimand.	Candidate L1/L4 mechanism, not proof of full chain.	Specific measures support only prespecified links.
Habit/automaticity (14)	Repetition and stable cues can automate responses.	Narrower than multilevel feedback and distal transition history.	Candidate contributor while requiring a controlled history effect.	Habit does not fully absorb β_history; otherwise novelty narrows.
Process-based psychotherapy (19)	Targets modifiable processes across diagnoses/schools.	Need not specify developmental distal-history effect.	Maps state-matched functions to primary Δβ_history and secondary fit/recovery tests.	Process change is temporal and not school-specific.

The residual contribution is a history-conditioned transition hypothesis. The model-specific empirical signature of regulatory path dependence is an incremental contribution of distal context–state–strategy–consequence–feedback history to a current transition after prespecified current-state, context, person, measurement-opportunity, and immediate-lag controls. It cannot be inferred from an adversity score, a diagnosis, high autocorrelation, current avoidance, a limited repertoire, or a single flexibility questionnaire.

Three predictions differentiate the proposed specification. First, compared with regulatory-flexibility models, a preregistered distal-history term should improve out-of-sample transition prediction after current flexibility, repertoire, state, context, traits, and immediate lag are included. Second, compared with the ACM, the joint history of strategy use and social or institutional feedback should add predictive value beyond current ecology and calibrated stress responsivity, especially under prespecified person–environment mismatch. Third, psychotherapy or accommodation should change a prespecified distal-history, context–strategy–outcome-fit, or recovery parameter in the predicted direction—not only symptoms. Internal and environmental interventions may change different parameters and need not be interchangeable.

The cited studies address several components and boundary conditions, but none tests the complete sequence from repeated regulatory strategy through path dependence to a specified health outcome and later recalibration. The full pathway is therefore a staged, falsifiable research program rather than an established causal mechanism. If history adds no stable predictive value beyond the closest alternatives, or if clinical improvement occurs without the proposed process changes, the model-specific claim must be narrowed or rejected.

The comparison also prevents semantic rescue. If “history” is operationalized only as current symptoms, adversity exposure, or retrospective beliefs, the test has reverted to established risk-factor models. If the recalibration label is assigned whenever treatment succeeds, it cannot discriminate among process accounts. Incremental value requires prospective or otherwise temporally resolved evidence that the proposed history and change parameters perform work that current flexibility, ecology, responsivity, and treatment-process accounts do not already perform.

## 4 The staged developmental co-calibration model

The theory is organized as six links rather than a single causal arrow. L0 specifies scope: biological dispositions, developmental exposures, current affordances, and stable person characteristics constrain but do not determine regulatory opportunities. L1 is the within-episode loop: current context and regulatory state shape strategy selection; immediate bodily, behavioral, and interpersonal consequences then update state and context. L2 concerns accumulation: repeated context–strategy–consequence–feedback sequences may change later selection and transition probabilities. L3 is the model-specific path-dependence claim. L4 concerns outcome-specific candidate pathways. L5 concerns change through psychotherapy, accommodation, or both.

Regulatory path dependence is the added predictive contribution of distal regulatory history after current context, current state, stable person characteristics, measurement opportunity, and immediate lag or inertia are controlled. Let Y_t denote the transition, C_t context, S_t regulatory state, T_i stable person characteristics, Y_t−1 the immediate lag, and H_t the prespecified distal history. The target is H_t in P(Y_t | C_t, S_t, T_i, Y_t−1, H_t). Its form and time window must precede analysis. Evidence comprises β_history and out-of-sample gain; prediction alone is not causal evidence. Nested models add stable traits (M1), immediate lag (M2), distal history (M3), history × mismatch (M4), and intervention-associated change (M5) to a current state/context model (M0). Dynamic structural equation modeling can estimate within-person temporal relations, although model choice depends on sampling and state structure (20).

Worked example. In a 14-day ecological momentary assessment burst with five assessments per day, let Y_t be a transition into withdrawal, C_t a prespecified high-interpersonal-demand context, S_t the current regulatory state, and Y_t−1 the immediately preceding transition. Define H_t before analysis as the proportion of high-demand episodes during the preceding seven days in which withdrawal was followed by short-term relief but no recovery within two assessments. M2 includes C_t, S_t, stable characteristics, measurement opportunity, and Y_t−1; M3 adds H_t. Regulatory path dependence is supported only if H_t yields replicable out-of-sample gain and a stable β_history. Alternative windows and operators are robustness analyses, not *post hoc* substitutes for a null primary definition.

This definition separates path dependence from adjacent constructs. Current regulatory inflexibility describes what a person does now; path dependence tests whether earlier trajectories add information about what happens next. Avoidance is a strategy category. Habit describes cue-dependent automaticity and may absorb part of β_history. Psychological flexibility is broader and often goal-oriented. Variability counts change but does not establish fit. State inertia concerns immediate persistence and is controlled in M2. A person can therefore be currently rigid without a detectable distal-history effect, or show comparable current flexibility while retaining different history-conditioned transition risks.

**Figure 1:**
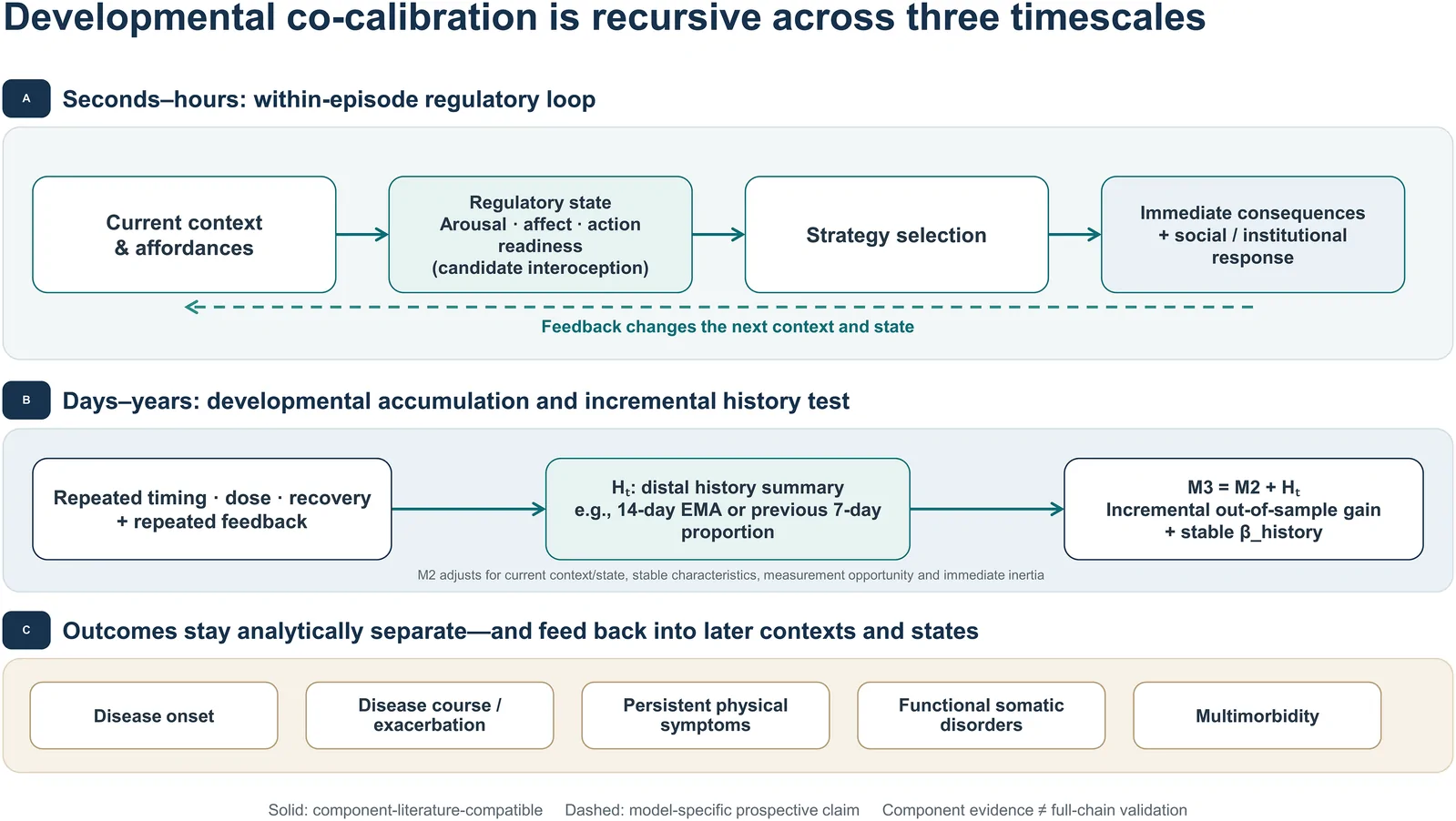
Developmental co-calibration as a recursive hypothesis across timescales. **(A)** Within episodes (seconds to hours), current context and affordances interact with a time-varying regulatory state, including candidate interoceptive inference processes, to shape strategy selection. Immediate consequences and interpersonal or institutional responses feed back into subsequent state and context. **(B)** Across repeated episodes (days to years), developmental timing, dose, recovery opportunity, and feedback may update transition probabilities. Regulatory path dependence is the incremental effect of prespecified distal history H_t on current transitions after accounting for current context, state, stable person characteristics, measurement opportunity, and immediate inertia. **(C)** Candidate associations are tested separately for disease onset, exacerbation or course, persistent physical symptoms, functional somatic disorders, and multimorbidity. Symptoms, care, medication, accommodation, validation, and stigma may feed back into later contexts and states. Solid arrows denote links compatible with component literatures; dashed arrows denote model-specific prospective hypotheses. Component evidence does not establish the full chain.

The chain is recursive. Within episodes, environmental responses may reinforce, punish, misread, or accommodate a strategy. Across episodes, these responses alter later affordances and expectations. Health symptoms, functional loss, medical treatment, medication, stigma, validation, and care pathways become new exposures that can modify subsequent context, state, and sampling. A developmental history may thus be partly produced by institutions, not only by the individual. **Figure 1** separates the within-episode loop, developmental accumulation, and outcome feedback.

Candidate mediators include allostatic or autonomic processes, interoception, health behavior, and feedback. Moderators include timing, controllability, mismatch, support, culture, sensory sensitivity, and medical vulnerability. Confounders include disease severity, stable neurodevelopmental or genetic differences, medication, socioeconomic conditions, prior care, access, measurement density, and common-method variance. Boundary conditions exclude autism or ADHD onset and psychogenic somatic disease and require repeated measurement that separates distal history from immediate inertia.

Pathways into and out of constrained dynamics need not be symmetric (21). Improvement may arise through new strategy learning, changed environmental demands, altered recovery, or reduced exposure without reversing the developmental path. Static trait language is therefore avoided. The model concerns probabilistic transition constraints that may persist yet remain modifiable.

## 5 Evidence for links and boundary conditions

The following literatures inform individual links, measurement choices, and boundary conditions; none validates the full chain.

Component compatibility cannot establish temporal direction, mediation, or the regulatory sequence producing an outcome association.

### 5.1 Developmental timing and biological embedding

Sensitive-period research shows that the effect of experience depends on developmental timing, circuit state, and environmental input (22, 23). Probabilistic epigenesis and cascade perspectives likewise make reciprocal developmental influence plausible (3, 4). Biological-embedding research describes routes through which adversity may become associated with later physiological and behavioral functioning (8). These literatures support L0–L2 and identify timing as a moderator. They do not demonstrate that repeated regulatory strategies create the specific distal-history parameter proposed here. Later plasticity also remains possible, so timing cannot be used as a deterministic explanation of persistence or treatment resistance.

Timing includes developmental stage, duration, repetition, predictability, recovery interval, and relational support. The same observable strategy can serve different functions across ages, while retrospective reports may not locate sequence onset. Prospective cohorts should link developmentally appropriate state/strategy measures to later intensive sampling rather than infer childhood transitions from adult rigidity.

### 5.2 Adversity, allostasis, and multimorbidity

Adverse childhood experience (ACE) studies report graded associations with later health risks (24, 25). Reviews also link ACE exposure with allostatic-load indices and multimorbidity (26, 27). Allostasis is relevant because it distinguishes short-term adaptation from cumulative cost (5, 6). However, ACE measures aggregate heterogeneous exposures, and allostatic load is not a single mechanism or biomarker. Threat, deprivation, loss, medical trauma, discrimination, and unpredictability may have different developmental implications (28).

For this model, adversity is an exposure and allostatic or autonomic change is a candidate mediator, not proof of path dependence. Multimorbidity is an outcome category requiring temporal and diagnostic specificity. Associations may reflect shared vulnerability, socioeconomic conditions, health behaviors, treatment access, reverse causation, or disease-related exposures. Observational evidence should therefore be described as compatible with L0 or L4, not as mediation of the full sequence.

The proposed history variable should also not be reduced to an ACE count. Two people with the same exposure score may have encountered different timing, controllability, buffering, strategy opportunities, and institutional responses. Conversely, similar regulatory histories may emerge from different exposures. Tests of co-calibration therefore require time-resolved strategy and feedback information in addition to adversity measures, while analyses of physiological pathways require temporal precedence and sensitivity to unmeasured confounding before mediation language is justified.

### 5.3 Neurodivergence, mismatch, and accommodation

Autism and ADHD are neurodevelopmental conditions, not defenses, trauma products, or regulatory strategies. Neurodiversity-informed approaches emphasize heterogeneity, environmental barriers, minority stress, and mutual misunderstanding (29–33). The model addresses a different question: how neurodevelopmental dispositions alter available affordances, regulatory costs, and the need for particular strategies in specific environments.

Camouflaging illustrates the distinction. Autistic people may camouflage to maintain participation or reduce stigma, while systematic reviews report associations with adverse mental health outcomes in many studies (34, 35). These associations do not show that camouflaging produces path dependence or somatic illness. Voluntariness, context, recovery, support, and measurement of current fit matter. The CAT-Q can index camouflaging-related behavior but cannot measure history dependence (36). Accommodation is not a secondary courtesy: changes in sensory, interpersonal, or institutional demands may reduce costs without requiring the person to abandon a necessary strategy (37). The clinical target is agency, fit, participation, and safety—not normalization.

If apparent “rigidity” changes when communication, sensory demands, predictability, or recovery opportunities change, a person-centered deficit explanation is weakened. Accommodation may be sufficient; persistence of an identity-congruent strategy is not pathology by definition.

### 5.4 Interoception, persistent symptoms, and respiratory illustration

Persistent physical symptoms (PPS) require an integrated formulation that recognizes bodily distress and disability without assuming a unitary cause (38). Active interoceptive inference offers a candidate explanation for how prior expectations, afferent signals, precision allocation, and action-dependent sampling may shape symptom appraisal and regulatory choices (15, 16). Developmental work suggests that interoceptive dimensions change across childhood and adolescence, but available measures remain heterogeneous (17, 18). Consequently, neither a questionnaire nor a physiological signal can be treated as a direct measure of developmental precision weighting or regulatory path dependence.

Respiratory symptoms provide an illustration, not a general proof. Dysfunctional breathing or breathing-pattern disorder can involve dyspnea and other symptoms in the absence of, or beyond, identifiable cardiopulmonary pathology (39–41). Disease and functional processes may coexist. Anxiety, autonomic activation, attention, breathing behavior, avoidance, airway inflammation, medication effects, and care responses can interact, but their contributions must be estimated rather than presumed. Research on stress and asthma is compatible with effects on exacerbation or course in susceptible individuals (42), while an association between ACEs and asthma does not by itself identify disease onset or mediation through regulatory strategies (43).

Five outcome classes must remain separate. Disease onset concerns incident pathology and requires disease-specific causal evidence. Exacerbation or course concerns changes in an established disease. PPS concerns persistent bodily symptoms irrespective of whether disease is present. Functional somatic disorders require their own diagnostic and mechanistic definitions. Multimorbidity concerns co-occurring long-term conditions and may reflect multiple shared or independent pathways. A signal in one class cannot be generalized to the others. Clinically, the implication is collaborative differential formulation: disease activity, functional processes, interoceptive threat, treatment effects, behavior, and environmental mismatch remain simultaneously thinkable until evidence permits narrowing.

### 5.5 Culture, institutions, and current person–environment fit

Cultural and institutional contexts influence which emotions and bodily expressions are legitimate, which strategies are rewarded, and which forms of support are accessible. Cross-cultural findings caution against assuming identical meanings or effects for suppression, reappraisal, or variability across settings (44, 45). Country labels are inadequate substitutes for individual orientation and local ecology.

Current person–environment fit is a comparator, not accumulated history. The same strategy may support participation in one setting and become costly in another. Co-calibration adds value only if prior strategy–feedback history predicts transitions beyond current fit. Accommodation, invalidation, diagnostic delay, and fragmented care should therefore be coded as possible moderators, exposures, or feedback—not background.

Current fit should be represented through person and environment components rather than a global satisfaction score alone. Context coding may include demands, sensory load, interpersonal power, available choice, and consequences of disclosure. Within-person designs can then test whether the same strategy has different consequences across contexts and whether distal history predicts residual differences, without attributing systemic constraints to individual inflexibility.

## 6 Therapeutic recalibration as a parameter-change hypothesis

Therapeutic recalibration is defined primarily as an intervention- or accommodation-associated change in the prespecified distal-history coefficient β_history. A favorable Δβ_history is interpretable only when baseline β_history predicted poorer context–strategy–outcome fit, functional cost, or delayed recovery; smaller history effects are not globally adaptive. Context–strategy–outcome fit and recovery are separate secondary process endpoints. Symptoms, functioning, participation, adverse effects, and patient-defined benefit remain clinical outcomes. Improvement in those outcomes without the primary process change remains clinically valuable but does not support this model-specific recalibration claim.

Psychotherapy and environmental accommodation are complementary routes. Psychotherapy may change state discrimination, expectations, strategy availability, action-dependent sampling, tolerance, or interpersonal learning; accommodation may alter demands, cues, feedback, recovery, and the need for costly strategies. Benefit may follow either route or both. Treating every improvement as recalibration would make the claim unfalsifiable.

Therapy-related neural or physiological findings should be interpreted modestly. Emotion-regulation research identifies distributed regulatory networks, and longitudinal imaging studies report therapy-associated functional changes in anxiety disorders (46–48). Memory-updating accounts offer plausible learning principles (49). None directly verifies the proposed developmental history parameter. Because onset and recovery may be asymmetric (21), treatment should not be portrayed as reversing development.

**Figure 2:**
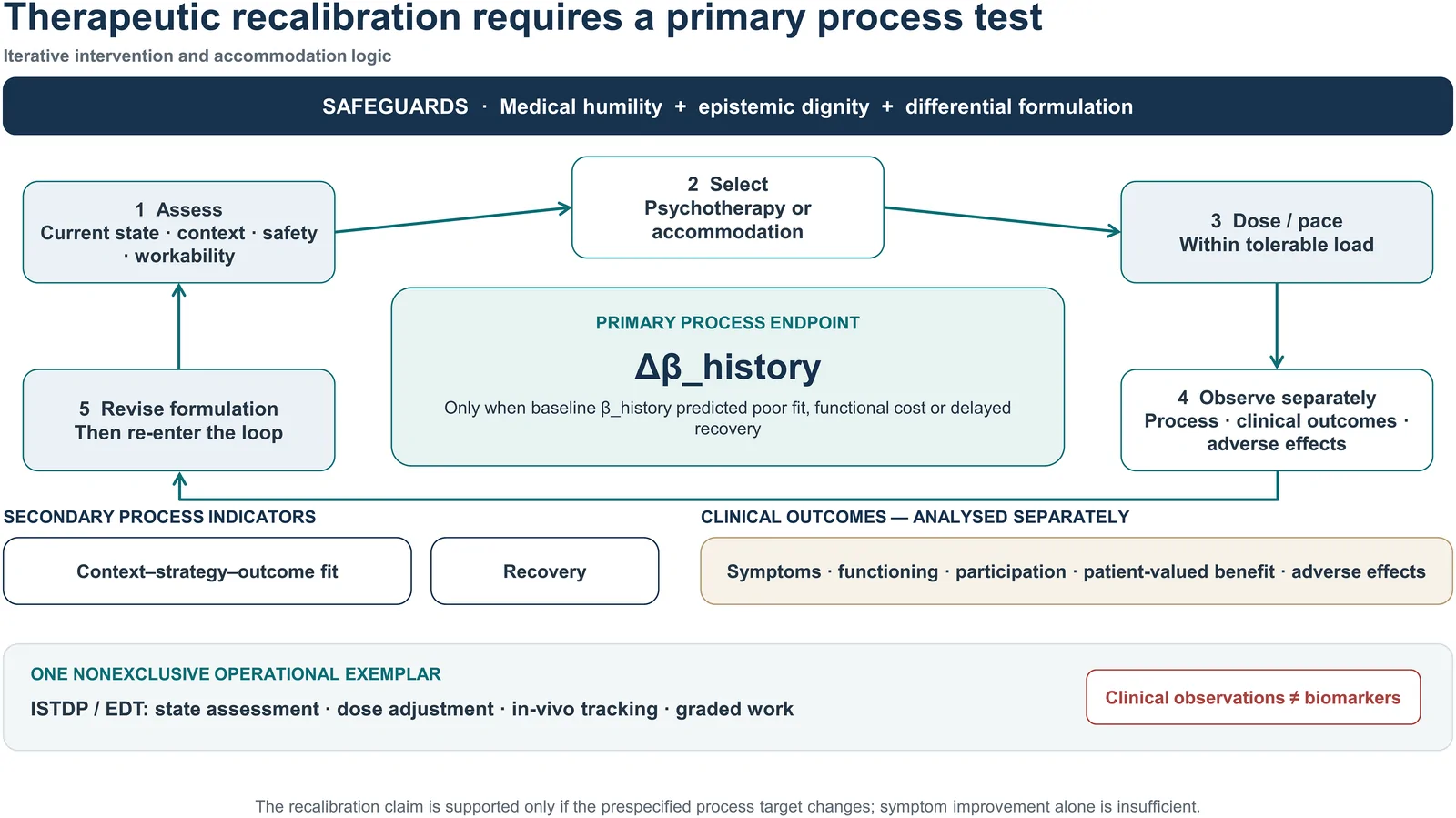
Therapeutic recalibration as an iterative, state- and context-sensitive hypothesis. Current state, context, safety, and workability are assessed before psychotherapy or accommodation is selected and titrated. Process indicators, clinical outcomes, adverse effects, fit, and recovery are observed separately and feed back into reassessment. The primary model-specific process endpoint is Δβ_history when baseline history predicted poorer context–strategy–outcome fit, functional cost, or delayed recovery. Changes in fit and recovery are secondary process endpoints; clinical benefit without Δβ_history remains valuable but does not support the primary recalibration claim. ISTDP and related EDTs are one nonexclusive example of state assessment, dose adjustment, *in-vivo* tracking, and graded work. Clinical observations are not direct biomarkers; medical humility and epistemic dignity remain cross-cutting safeguards.

**Figure 2** represents an iterative assess–select–dose–observe–revise loop. Current state, context, safety, and workability are assessed before an intervention or accommodation is selected and titrated. The primary model-specific process endpoint is Δβ_history under the prespecified baseline condition; context–strategy–outcome fit and recovery are secondary process endpoints. Clinical outcomes and adverse effects remain separate. All observations feed back into reassessment; clinical indicators are not direct biomarkers.

A credible intervention test requires repeated baseline, treatment, and follow-up measurement. Comparable context coverage and measurement invariance are necessary; otherwise, Δβ_history may reflect exposure or measurement change. Expectancy, regression to the mean, concurrent care, and selective dropout remain alternatives. Confidence increases when process change precedes patient-defined functional benefit and generalizes beyond treatment.

## 7 General requirements for psychotherapy and accommodation

The model proposes process requirements, not a new therapy package. Clinicians first assess current regulatory state, context, safety, affordances, and recovery before selecting an intervention. The formulation remains provisional and must change when medical, behavioral, or contextual data conflict with it.

Second, formulation should identify the current function of a regulatory strategy. Withdrawal, compliance, masking, certainty-seeking, intellectualization, avoidance, or overcontrol may reduce threat, protect belonging, prevent overload, or preserve agency. Function is context-dependent; the same form can serve different functions, and the same function can be served by different forms.

Third, intervention dose should be titrated to learning opportunity rather than intensity. Activation that is too low may not engage the target process; activation that exceeds reflective or physiological workability may produce flooding, dissociation, somatic escalation, compliance, or dropout. State-dependent workability and safety-gated microdecisions provide one way to formalize this principle (50, 51), but the requirement is not therapy-school specific.

Fourth, track context/state → strategy → immediate consequence → feedback → updated state/context *in vivo*. Fifth, test corrective learning: patients encounter relevant cues, try an alternative response, evaluate consequences, and transfer learning. Alliance enables this work and supplies feedback but does not replace process specification (52).

Sixth, transfer and accommodation should be planned explicitly. Repertoire expansion is unhelpful if environments continue to punish its use or if symptoms and care demands prevent recovery. Seventh, review should separate process indicators from clinical outcomes. Fit, recovery, distal-history effect, symptoms, functioning, participation, adverse effects, and dropout should not be collapsed. **Table 3** maps these requirements to one ISTDP/EDT example and states the corresponding safety and evidence boundary.

**Table 3:** General psychotherapy requirements and ISTDP/EDT as one operational exemplar.

General requirement	Observable process function	ISTDP/EDT example	Safety/evidence boundary
State and context assessment	Identify current state, affordances, safety, and plausible contingencies.	Moment-to-moment anxiety/defense/affect monitoring and collaborative formulation.	Observation is fallible; reassess; maintain medical evaluation.
Functional formulation	Specify what a strategy accomplishes now and where costs arise.	Clarify protective function before inviting change.	No universal maladaptive defense; context and voluntariness matter.
Dose titration	Adjust intensity, pacing, and task to tolerance and learning opportunity.	Graded pressure/challenge with monitoring and regulation.	Avoid flooding/coercion; adverse effects and dropout are outcomes.
*In-vivo* process tracking	Link state → strategy → consequence → feedback within episodes.	Track bodily cues, affect, action tendency, and interpersonal response.	Clinical sequences are hypotheses, not neural/physiological readouts.
Corrective learning	Test alternatives and update expectations under adequate safety.	Experiential work and revised interpersonal prediction.	Mechanism requires temporal/process evidence, not *post hoc* narrative.
Transfer and accommodation	Generalize gains and/or change environmental demands and supports.	Between-session practice plus workplace/family/sensory accommodation.	Person change is not always indicated; environment-only effects are relevant.
Review and revision	Track fit, recovery, function, symptoms, safety, and prespecified distal-history effect.	Collaborative outcome review and reformulation.	No symptom-only proof of recalibration; reject mechanism if process prediction fails.

State-dependent and safety-gated models imply a portable extension of dose regulation (50, 51). Where feasible, patients should increasingly recognize early signs of overload or collapse, communicate limits, pause or sequence tasks, seek accommodation, and re-enter emotionally relevant work when sufficient capacity returns. This is a model-derived clinical proposal rather than established ISTDP-specific mechanism evidence. Autonomous self-pacing is not avoidance by definition: its function depends on whether it preserves workable engagement and recovery or becomes a context-insensitive restriction.

Safety monitoring extends beyond crisis events. Worsening bodily symptoms, dissociation, compliance, loss of functioning, treatment avoidance, or invalidation may indicate dose or formulation mismatch and should trigger reassessment. Patient-defined goals and workability reports therefore accompany clinician observations and standardized outcomes.

## 8 ISTDP and related EDTs as one operational exemplar

Intensive Short-Term Dynamic Psychotherapy (ISTDP) and related Experiential Dynamic Therapies (EDTs) remain one operational exemplar, not the model itself. They are relevant because their clinical vocabulary makes state assessment, anxiety-channel monitoring, affect tolerance, defense or strategy observation, graded intervention, and dose adjustment explicit (53–55). These procedures can instantiate parts of the assess–dose–safeguard–review loop, but their use does not demonstrate regulatory path dependence or a school-specific or neurobiological recalibration claim.

The outcome evidence also requires proportionate wording. Meta-analytic work supports the efficacy of EDTs across randomized studies while reporting heterogeneity and continuing process-research needs (56). Reviews of ISTDP and short-term psychodynamic treatment for functional somatic disorders report promising effects alongside methodological limitations (57, 58). These findings justify clinical relevance, not universal superiority. Other psychodynamic, cognitive-behavioral, exposure-based, acceptance-based, mentalization-based, systemic, body-oriented, and process-based interventions may implement comparable functions through different procedures.

For fragile, dissociative, somatically reactive, or neurodevelopmentally overloaded patients, fidelity to the present framework means increasing support, pacing, integration, predictability, and accommodation when required. Direct pressure, challenge, or affective mobilization is not inherently more recalibrating. Clinical observations of bodily anxiety or defensive shifts are hypotheses about process and should not replace medical assessment or be described as biomarkers. Model validity must therefore be tested through school-independent functions and process parameters rather than brand comparisons.

The exemplar can be evaluated without assuming that its school-specific constructs are correct. Session coding could identify when state assessment changes the selected intervention, whether dose is reduced after signs of overload, whether the patient increasingly initiates pacing, and whether alternative responses are followed by recovery and transfer. Comparable codes can be applied to other therapies. A functional comparison would ask which procedures produce the hypothesized state–strategy–feedback changes for which patients, not whether one school uses the preferred terminology.

An evidence ladder separates observation, repeated process coding, experimental or quasi-experimental manipulation, and clinical outcomes. Neural or autonomic measures may add convergence but cannot independently confirm a therapist’s interpretation. This hierarchy permits mechanistic hypotheses without overstating ISTDP-specific evidence.

## 9 Testable predictions and falsification criteria

The model is testable only when its links, comparators, and null results are prespecified. Intensive longitudinal data are central because path dependence concerns transitions and history, not retrospective narratives alone. Sampling density must match the proposed timescale; missingness, measurement reactivity, differential exposure opportunity, and stable between-person differences require explicit modeling. Dynamic structural equation, hidden-Markov, state-space, or other transition models may be appropriate depending on whether states are continuous or discrete (20).

Test 1 examines the within-episode loop. Current context and state should predict strategy selection, and immediate consequences or environmental responses should predict subsequent state/context within persons. Failure to obtain directional or out-of-sample improvement over contemporaneous associations weakens L1.
Test 2 examines accumulation. Prespecified features of repeated context–strategy–feedback sequences should add prediction of later selection probabilities after stable traits and immediate lag are included. A null or nonreplicating history term weakens L2 and prevents a path-dependence interpretation even if adversity and symptoms are associated.
Test 3 is the primary model-specific test. M3, containing distal history, should improve out-of-sample transition prediction over M2, which already contains current state, current context, stable characteristics, current flexibility, diagnosis, symptom severity, measurement opportunity, and immediate inertia. β_history should be stable across reasonable specifications and replicated. If current flexibility, habit, diagnosis, or severity absorbs the effect, the claimed novelty narrows accordingly.
Test 4 examines mismatch. A preregistered distal-history × current-mismatch interaction should identify contexts in which the history association is stronger, while safety, support, or accommodation may attenuate it. *Post hoc* context coding or an interaction that does not replicate cannot support the claim.
Test 5 examines outcome specificity. Associations and candidate mediations should be estimated separately for disease onset, disease exacerbation or course, PPS, functional somatic disorders, and multimorbidity. A result created only by pooling heterogeneous outcomes weakens rather than broadens the model. Temporal precedence, disease severity, medication, socioeconomic conditions, care access, and reverse causation require attention.
Test 6 examines path asymmetry. Models that estimate entry into and recovery from constrained dynamics separately should outperform a forced-symmetry account if onset and recovery differ (21). If a symmetric model is sufficient and replicates, the asymmetry claim is unsupported.
Test 7 examines therapeutic recalibration. The primary model-specific process test asks whether psychotherapy and/or accommodation changes the prespecified β_history in the predicted direction before or alongside clinical outcomes, provided baseline history predicted poorer fit, functional cost, or delayed recovery. Secondary tests examine context–strategy–outcome fit and recovery separately. Randomized, adaptive, interrupted time-series, or sufficiently dense idiographic designs may be used. Changed context exposure and measurement invariance must be modeled. Clinical improvement without Δβ_history remains valuable but does not support the primary recalibration claim; a process change without temporal relation to functioning, participation, symptoms, safety, or patient-defined benefit lacks clinical value.

**Table 4** consolidates these tests. Failure of the preregistered history term to improve out-of-sample prediction would weaken the model-specific claim even if component associations were replicated. The theory should therefore be revised or abandoned if history, mismatch, outcome specificity, and parameter-change predictions do not add reproducible value.

**Table 4:** Preregisterable tests and results that would weaken the model.

Staged link	Preregistered prediction	Design/estimand	Comparator/confounders	Result that weakens the model
L1 Episode loop	State/context predict strategy; consequences/feedback predict subsequent state/context.	Intensive multimodal time series; lagged within-person estimates.	Time trend, reactivity, reverse lag.	No directional or out-of-sample gain over contemporaneous association.
L2 Accumulation	Repeated context–strategy–feedback sequences alter later selection probabilities.	Prospective sequence features; M1/M2 versus M3.	Stable preferences, exposure opportunity, immediate inertia.	Distal sequence adds no stable incremental prediction.
L3 Path dependence	β_history remains after current state/context, traits, flexibility, diagnosis, severity, and immediate lag.	Preregistered history operator; DSEM/HMM/Markov; replication.	Habit, current fit, baseline severity, measurement opportunity.	β_history ≈ 0, unstable, nonreplicating, or no out-of-sample gain.
L3 × mismatch	The distal-history association is stronger in prespecified mismatch/threat contexts and weaker with safety/accommodation.	M3 versus M4 interaction; stratified robustness.	Differential exposure/measurement; *post hoc* context coding.	No interaction or opposite direction across replications.
L4 Outcome specificity	Links differ for onset, course/exacerbation, PPS, functional somatic disorders, and multimorbidity.	Outcome-specific cohorts/models; temporal precedence.	Reverse causation, diagnosis error, care access, medication.	Effects exist only after heterogeneous pooling or vanish with severity controls.
L5 Path asymmetry	Entry into and recovery from constrained dynamics require different parameters.	Compare symmetric and asymmetric dynamic models.	Unequal observation windows and intervention timing.	A symmetric model is sufficient and replicates.
L5 Recalibration	Primary: therapy/accommodation changes prespecified β_history under the baseline risk condition before/alongside clinical outcomes; fit and recovery are secondary.	RCT/SMART/ITS/N-of-1; time-varying parameter or phase comparison.	Regression to the mean, concurrent care, expectancy, attrition, context exposure, and measurement noninvariance.	Clinical improvement without Δβ_history, or Δβ_history unrelated to outcomes; secondary process changes cannot rescue a null primary test.

Robustness should be tested across plausible history operators, lags, and state definitions without selecting the strongest effect. Confirmatory analyses should separate within- from between-person associations, report calibration and prediction, and use temporal or external validation where feasible. Qualitative data may clarify function and context but cannot replace transition tests.

## 10 Limitations and ethical-methodological safeguards

The framework is a probabilistic research program, not a developmental causal chain. Observational arrows do not establish mediation. Dense prospective measurement is needed, and estimates may depend on timescale, state definition, and missingness. Interoception is multidimensional; allostatic load is not a unitary biomarker; cultural categories may conceal local variation; and process coding may reflect allegiance or common-method bias. Model-specific terms should be removed if they add no discrimination or prediction.

Medical humility is non-negotiable. Autism and ADHD are not explained as regulatory adaptations; adversity does not inevitably cause disease; anxiety does not become asthma; and treatment response does not prove psychogenic etiology. Disease onset, disease course, PPS, functional somatic disorders, and multimorbidity require separate evidence and differential diagnosis. Organic pathology, medication effects, functional processes, interoceptive threat, behavior, and environment may coexist.

Epistemic dignity is an ethical and methodological safeguard (59, 60). Patients’ embodied, affective, relational, and contextual reports should be treated as potentially valid data and triangulated with behavior, history, medical assessment, physiology, and environmental constraints. This stance protects against both dismissing symptoms as “merely psychological” and granting neural language automatic authority. It does not make subjective reports infallible. Clinically, the formulation and dose must be revised when the patient’s experience, body, functioning, safety, or context indicates mismatch.

Finally, the new terminology creates its own risk. A model that can retrospectively label any stable pattern as path-dependent and any helpful change as recalibration would reproduce the umbrella problem it was designed to solve. Researchers should therefore publish null history effects, retain simpler comparator models when they perform equally well, and avoid adding qualifiers merely to preserve the theory. Ethical safeguards and methodological parsimony point in the same direction.

## 11 Discussion and conclusion

Developmental co-calibration is presented as an integrative and testable specification, not as a claim that reciprocal development, allostasis, flexibility, habit, interoception, or process-based treatment are new. Its residual contribution is regulatory path dependence: prior regulatory history must add reproducible information about current transitions beyond current state, context, flexibility, stable characteristics, diagnosis, symptom severity, and immediate inertia.

The full sequence remains unvalidated. Evidence for individual links motivates a staged research program, not a general causal account of chronic disease. Health claims remain outcome-specific. Psychotherapy or accommodation supports the model-specific recalibration hypothesis only when the prespecified primary distal-history parameter changes under its baseline condition; fit and recovery are separate secondary process endpoints.

This narrower formulation preserves clinical usefulness. It directs attention to state, strategy function, tolerable dose, feedback, recovery, environmental affordances, and differential formulation. ISTDP/EDT provide one operational illustration, while model validity depends on school-independent tests. The framework earns its place only if these tests improve prediction, safety, participation, or treatment selection; otherwise its model-specific terminology should be reduced.

The intended contribution is disciplined integration: a clinically recognizable sequence translated into explicit variables, competing models, and disconfirming results. This preserves the neurobiopsychosocial red thread without substituting breadth or plausibility for evidence.
